# Prostate-Specific Membrane Antigen-Positron Emission Tomography-Guided Radiomics and Machine Learning in Prostate Carcinoma

**DOI:** 10.3390/cancers16193369

**Published:** 2024-10-01

**Authors:** Justine Maes, Simon Gesquière, Alex Maes, Mike Sathekge, Christophe Van de Wiele

**Affiliations:** 1 Department of Nuclear Medicine, AZ Groeninge, 8500 Kortrijk, Belgium; justinemaes@hotmail.com (J.M.); alex.maes@azgroeninge.be (A.M.); 2 Department of Nuclear Medicine, University Hospital Ghent, 9000 Ghent, Belgium; simon.gesquiere@uzgent.be; 3 Department of Morphology and Functional Imaging, University Hospital Leuven, 3000 Leuven, Belgium; 4 Department of Nuclear Medicine, University of Pretoria, Pretoria 0002, South Africa; mike.sathekge@up.ac.za; 5 Department of Diagnostic Sciences, University Ghent, 9000 Ghent, Belgium

**Keywords:** PSMA, prostate carcinoma, radiomics

## Abstract

**Simple Summary:**

Available studies suggest that radiomics and machine learning applied to PSMA-radioligand avid primary prostate carcinoma have potential to serve as an alternative for non-invasive Gleason score characterization, for the prediction of biochemical recurrence and to differentiate benign from malignant increased tracer uptake. However, prior to their implementation in clinical practice, additional, clinically relevant studies performed according to recently published guidelines and checklists, offering full transparency, including large enough datasets as well as external validation, are mandatory.

**Abstract:**

Positron emission tomography (PET) using radiolabeled prostate-specific membrane antigen targeting PET-imaging agents has been increasingly used over the past decade for imaging and directing prostate carcinoma treatment. Here, we summarize the available literature data on radiomics and machine learning using these imaging agents in prostate carcinoma. Gleason scores derived from biopsy and after resection are discordant in a large number of prostate carcinoma patients. Available studies suggest that radiomics and machine learning applied to PSMA-radioligand avid primary prostate carcinoma might be better performing than biopsy-based Gleason-scoring and could serve as an alternative for non-invasive GS characterization. Furthermore, it may allow for the prediction of biochemical recurrence with a net benefit for clinical utilization. Machine learning based on PET/CT radiomics features was also shown to be able to differentiate benign from malignant increased tracer uptake on PSMA-targeting radioligand PET/CT examinations, thus paving the way for a fully automated image reading in nuclear medicine. As for prediction to treatment outcome following ^177^Lu-PSMA therapy and overall survival, a limited number of studies have reported promising results on radiomics and machine learning applied to PSMA-targeting radioligand PET/CT images for this purpose. Its added value to clinical parameters warrants further exploration in larger datasets of patients.

## 1. Introduction

Radiomics allows the extraction of quantitative parameters (radiomic features) from segmented tumor lesions from medical images that cannot be appreciated visually [1,2,3,4]. Radiomic feature extraction methods include the assessment of global tumor properties from individual voxels, from the gray-level histogram, from the shape of the lesion studied, as well as second-order statistics. Second-order statistics include the gray-level co-occurrence matrix (GLCM), which assesses how often gray levels occur together in neighboring pixels within an image; the gray-level run length matrix (GLRM), which quantifies lengths in number of pixels with the same gray-level value; the neighborhood grey-level difference matrix (NGLDM); which assesses differences of gray levels between one voxel and all of its directly neighboring voxels in three dimensions; and the gray-level zone length matrix (GLZLM), which provides information on the size of homogeneous zones for each gray level in three dimensions. Using available software radiomics algorithms, some of which are freely available, e.g., LIFEx version 7.6.0 and PyRadiomics version 3.1.0, a large number of features are often extracted, many of which are highly correlated (multicollinearity) and thus redundant and need to be removed for further analysis. Approaches used to this purpose include, amongst others, collinearity analyses based on Pearson and intra-class correlation analyses, algorithm-based feature selection, and principal component analyses. The remaining features, split up in a training and validation set, usually then serve as input for machine learning. To date, the most frequently used type of machine learning in medical imaging, related to usually limited available datasets, is supervised learning, which requires a dataset that includes inputs and correct outputs, allowing a model to learn over time and to minimize its error. Typical supervised learning algorithms are, e.g., linear and logistic regression; support vector machines (SVMs), which construct a hyperplane where the distance between two datasets is at its maximum; K-nearest neighbor (KNN), which calculates the distance between data points and assumes that similar points can be found near each other; and random forest (RF), which merges uncorrelated decision trees reducing variance, thus creating more accurate data predictions [5,6].

Prostate carcinoma is one of the most malignant cancers worldwide and the fifth leading cause of cancer death [7]. Positron emission tomography using radiolabeled prostate specific membrane antigen targeting PET-imaging agents such as ^68^Ga-PSMA-11, ^18^F-PSMA-1007, and ^18^F-DCFPyl have been increasingly used over the past decade for imaging and directing prostate carcinoma treatment [8,9]. Here, we summarize the available literature data on radiomics and machine learning using these imaging agents in prostate carcinoma. A search was performed on PubMed using the search terms “prostate specific membrane antigen”, “PSMA”, “folate hydrolase” in conjunction with “prostate”, “carcinoma”, “cancer”, “radiomics”, “radiomic”, and “machine learning”. The methodological quality of the studies with clinical relevance end-points was assessed using the recently proposed METhodological RadiomICs Score (METRICS) [10,11]. METRICS includes 30 items within various categories that were accorded different weights with a consensus threshold of 75% by a group of 59 international radiomics experts. In descending order of importance, the categories are study design, imaging data, image processing and feature extraction, metrics, and comparison and testing. METRICS classifies the quality of radiomics studies as very low, low, moderate, good, or excellent.

## 2. Methodological Studies

Image reconstruction, tumor or lesion segmentation, and preprocessing are well-known parameters influencing radiomic feature values and stability. Furthermore, different PSMA-targeting PET ligands have been reported to have different IC50 values, internalization rates, and blood clearance rates, which may result in different feature values and hamper their mixed use in clinical practice. Some of these issues have been addressed by a number of authors (see Table 1). Fooladi et al. studied the impact of penalized likelihood (Q.Clear) and ordered subset expectation maximization (OSEM) reconstruction on the robustness of 129 radiomic features derived from 30 ^68^Ga-PSMA-11 avid metastatic prostate carcinoma lesions (eight patients) situated in the abdominal and pelvic region using the LIFEx software version 7.6.0 and a threshold of 41% of SUVmax for lesion segmentation [12]. For the OSEM reconstruction method, 15 s, 30 s, 1 min, 2 min, 3 min, 4 min, and 5 min images were studied and for the Q.Clear reconstruction mode, Beta values between 100 and 700, at intervals of 100 and a duration of 2 min, were studied. When considering the Q.Clear reconstruction method, only 23% showed a very small (COV < 5%) or small variation (COV 5% < COV < 10%) across changes in beta value. Inversely, when considering OSEM, more than half of the radiomic features showed a very small or small variation across the changes in duration. Of interest, none of the morphological features were found to have a small or a very small variability. Pasini et al. studied the robustness of radiomics to variations in segmentation methods (manual, region growing, and thresholding (cut-offs used were not specified)) of 78 ^18^F-PSMA-1007 avid primary prostate carcinoma lesions (46 low-grade and 35 high-grade lesions) [13]. A total of 1781 radiomic features were extracted for the three datasets using PyRadiomics, 107 extracted from the original images, 744 from wavelet decomposed images, and 930 from Laplacian of Gaussian (LoG) filtered images. Jaccard indices for the paired-wise comparison of the three segmentation methods varied from 0.51 to 0.58. Shape features were proven to be the least reproducible (average intraclass correlation coefficient (ICC): 0.27), and GLCM features were the most reproducible (ICC: 0.89). Furthermore, segmentation methods negatively impacted the wavelet-decomposed images (ICC range: 0.49–0.56 for the different features subgroups studied). The authors also assessed the impact of the lesion segmentation method on the accuracy of six machine-learning models (LDA, SVM, KNN, RF, AdaBoost, and NN classifiers) for separating low-grade and high-grade lesions. The region-growing-LDA model was found to be the most accurate (AUC 79.2%). However, all models were based on retained/selected wavelet features that showed the worst average ICC performance. Dutta et al. assessed the robustness of radiomic features extracted from malignant involved prostates of 142 patients as defined by ^68^Ga-PSMA-11 PET images on automated prostate volume delineation, applying the publicly available nnU-Net model on simultaneously obtained T2 MRI images on the one hand and the manually delineated clinical target volume (CTV) on the other hand [14]. The mean Dice coefficient (DC) between the CTV and the nnU-Net segmentation was 0.78 (range 0.02–092). To ensure significant spatial overlap, only patients with a DC > 0.6 were used for analysis. Out of 1037 features extracted, 51 had an ICC > 0.9, and 148 > 0.75 and <0.75. Approximately 88% of these features were wavelet-based and consisted mainly of grey-level texture features. Kendrick et al. prospectively assessed inter- and intra-tracer robustness of radiomic features in 18 metastatic prostate cancer patients that were randomized to one of three rest–retest groups, intra-tracer ^68^Ga-PSMA-11 PET (five patients), intra-tracer ^18^F-PSMA-1007 PET (three patients), and inter-tracer between ^68^Ga-PSMA-11 and ^18^F-PSMA-1007 PET (eight patients) [15]. Test–retest scans were taken within 2–7 days from each other. Patient lesions were delineated manually, followed with a baseline global 3 SUVbw threshold applied to the PET images. Only lesions > 1.5 cm^3^ were used for analysis, and 107 features were extracted from each lesion using PyRadiomics. For both intra-tracer groups, the number of features extracted rated as excellent (ICC > 0.9) or good (0.75 < ICC < 0.9) were comparable (69% for ^68^Ga-PSMA-11 and 66% for ^18^F-PSMA-1007), with ^68^Ga-PSMA-11 having a higher percentage of excellent features relative to ^18^F-PSMA-1007 (50% versus 38%). The most stable features identified were first-order statistics and entropy-based features from the GLCM feature family. Applying filters to the data did not improve their overall repeatability. Inversely, for the inter-tracer group, only 9% of features were classified as either excellent or good. Finally, Werner et al. assessed repeatability of 29 radiomic features derived from 230 lesions (177 bone, 38 lymph nodes, and 15 others) visualized on ^18^F-DCFPyl in 21 patients in a test–retest setting (PET scan acquired twice in each patient within 7 days) [16]. Segmentation of the lesions was performed manually, and features were extracted using the Interview software (the mean value of the Dice coefficient was 0.48 (range 0.03–0.99)). An acceptable reproducibility was found for the entropy and homogeneity (within subject coefficient of variation (wCOV) of 16% and 12.7% respectively); for the remaining 27 features, the wCOV found were >21.7%.

## 3. Primary Prostate Carcinoma Discrimination and Characterization

For the purpose of prostate carcinoma diagnosis, transrectal ultrasound-guided biopsy (TRUS-GB) suffers from potential sampling error and interobserver variability; leads to the over-detection of clinically insignificant cancer; and may cause procedure-related bleeding, pain, and infection [17,18]. While multiparametric MRI may make it possible to avoid a biopsy in up to one-third of patients, it suffers from a low specificity in very-low-risk patients and from a poor sensitivity when confronted with small intraprostatic tumors, especially when situated centrally. Furthermore, in spite of the introduction of the PI-RADS version 2 scoring system, mp-MRI inter-reader reproducibility is suboptimal, and ISUP (International Society of Urological Pathology) upgrading is reported in approximately 30% of mp-MRI-based biopsies [19]. PSMA-targeted PET imaging has a higher sensitivity and specificity for the detection of primary prostate carcinoma when compared to MRI, and limited results suggest that it may aid in clinical decision-making by eliminating the further need for biopsies. However, in spite of this, in up to 10% of patients presenting with increased PSA levels, primary tumor lesions remain undetected on PSMA-targeted PET/CT, and up to 40% of these lesions are subsequently shown to be clinically significant (ISUP > 1) [13,14]. Furthermore, prostate carcinoma is renowned for its multifocality. In a study by Mouraviev et al. analyzing 947 prostatectomy specimens, 78% of specimens contained multifocal prostate carcinoma with, on average, 2.24 lesions being identified in these specimens [20]. When considering a safe implementation of focal therapies, accurate assessment of the intraprostatic local disease extent is of paramount importance. Accordingly, the authors studied the potential of radiomics applied to PSMA-targeting PET/CT examinations to identify and characterize prostate tracer-avid lesions and to predict the existence of visually undetectable intraprostatic carcinoma (see Table 2).

### 3.1. ^68^Ga-PSMA-11

Zamboglou et al. studied a prospective cohort of 20 patients and a retrospective validation cohort of 40 patients suffering from biopsy-proven prostate carcinoma scheduled to subsequently undergo radical prostatectomy and lymphadenectomy [20]. Three-dimensional histologic assessment of the radical prostatectomy specimen was used as the gold standard. Radiomics analysis was performed on ^68^Ga-PSMA-11 PET/CT images resampled to obtain 2 × 2 × 2 mm^3^ voxels obtained prior to prostatectomy using an in-house MATLAB- based software. For analysis, the entire prostate volume, defined using CT, was subtracted by the tumor volume, assessed in three different manners, respectively, using SUV-window leveling, a 40% threshold of SUV-max, and 3D histology. Radiomic features were subsequently extracted from the three tumor volumes as well as non-tumor volumes (subtraction volumes between the prostatic gland on CT scans and the three respective tumor volumes). A wavelet band-pass filtering, as well as an equal-probability quantization algorithm, were applied on the voxel intensities within the contours. As anticipated, SUV-related features proved significantly different between PCa and non-PCa tissue. However, the best-performing RF to differentiate between GS 7 and GS > 7 (pooled cohorts), as well as between pNO and pN1 status, was the TF quantization algorithm + short zone high gray-level emphasis (QSZHGE). Of interest, in their study, median volumes of GTV−40% proved significantly smaller than median GTV histology volumes.

Zamboglou et al. subsequently reported on the same prospective cohort of 20 patients and an external validation cohort of 52 patients from Nanjing, China, using a similar study set-up, however, focusing solely on the prostate volume subtracted by the gross PET-visible tumor volume, aiming to identify features that allowed identification of additional microscopic sites of tumor involvement to the visible primary [21]. In this study, the software PyRadiomics was used, and 152 features were extracted. A locally binary pattern filter (LBP) was applied. Features that proved significantly different on visually non-PCa-PET images that did or did not contain histologic prostate carcinoma were respectively size-zone non-uniformity normalized (SZNUN), and small area emphasis (SAE) with ROC AUC values of 0.8 were found for both features. In the training cohort, visual image interpretation missed 134 lesions, with a median diameter of 2.2 mm, in 12 patients, 75% of which had clinically significant PCA (ISUP > 1). In the validation cohort, PCa was missed in 26 patients, 77% of which possessed clinically significant PCa. In the validation cohort, the sensitivities of LBP-SZNUN and LBP-SAE were ≥0.8.

Yi et al. studied 100 patients that were referred for ^68^Ga-PSMA-11 PET/CT imaging to exclude primary prostate carcinoma in whom imaging proved negative and in whom imaging results were subsequently shown to be truly or falsely negative through biopsy or long-term follow-up [22]. Data from 64 patients, of which 39 proved malignant, were used as the training set, whereas data from the remaining 36 patients, of which 21 proved malignant, were used as validation set. The prostate was manually segmented into the right and left lobe on the PSMA PET images using the corresponding CT scan, and 1781 features extracted on both early and delayed PET images. Using the minimum redundancy maximum relevance method, the 10 best-performing features, one first-order feature, three wavelet-based features, four Gaussian-based features, and one logarithm based feature were selected and used to create three random forest machine learning models, one based on early PET images, one on delayed PET images, and one on both standard and delayed images. AUC values of the radiomics RF models proved significantly higher than those of PSA density (0.903, 0.856 and 0.925 versus 0.662, respectively, *p* = 0.007, *p* = 0.045 and *p* = 0.005). 

Ghezzo et al. retrospectively studied 43 patients suffering from prostate carcinoma in whom baseline ^68^Ga-PSMA-11 PET/CT was performed and that had diagnostic biopsy (biopsy ISUP grades) and full radical prostatectomy (post-surgical ISUP grade) performed as the sole therapy [23]. The whole prostate was manually segmented on co-registered PET/CT images and used for radiomics feature extraction (a total of 103 IBSI compliant RFs were extracted) using PyRadiomics 7.6.0 following resampling to 2 × 2 × 2 mm^3^ voxels. SUVmean values were also obtained using a 41% threshold of SUVmax. The sample size was split into four training sets and one validation set using stratified cross-validation and oversampling where necessary, yielding a 1:1 proportion for the various ISUP classes. Logistic regression, support vector machine, and K-nearest neighbor classifiers were trained to predict psISUP. The most relevant non-redundant features extracted, defined using the minimum redundancy maximum relevance algorithm, proved to be the GLSZM—zone entropy, Shape—least axis length, first-order—minimum, and GLSZM—small area low gray-level emphasis. The best performing model, using LR and GLSZM—zone entropy and shape—least axis length as RFs, had a balanced accuracy of 87.6% versus 85.9% for biopsy. Biopsy underestimated the aggressiveness of PCa in nine patients in whom ISUP < 4 was later revised to be high-grade PCA. Using a similar study-set up, Solari et al. studied 101 patients using ^68^Ga-PSMA-11 PET/MRI, and 107 features were derived from PET and MRI images [24]. Prostate segmentation was manually performed on MRI images and using the Fuzzy Locally Adaptive Bayesian segmentation tool for PET. In addition to the aforementioned features, six SUV-tumor based features were also included. Using SVM, the best-performing model was the one that combined features from both highest single modalities (PET + ADC, bACC: 82 ± 5%).

### 3.2. ^18^F-PSMA-1007

Yao et al. studied 173 histologically confirmed primary prostate carcinoma patients who underwent radical prostatectomy without adjuvant therapy prior to ^18^F-PSMA-1007 PET imaging [25]. Tumor volumes of interest were generated using thresholds of 30%, 40%, 50%, and 60% of the SUVmax value. Radiomic features were obtained from these volumes using the LIFEx software following voxel resampling to a size of 2 × 2 × 2 mm^3^. Features were ranked using the mRMR method while minimizing the correlation between features, and the top 10 GS-associated RFs were selected for each thresholding VOI. Prediction models for ISUP ≥ 3 were constructed using SVM and optimal RFs as well as PSA levels. The best predictive performance for ISUP-grade assessment as well as for the presence of vascular invasion in training and testing cohorts were obtained using the 50% SUVmax model (AUC 0.82 and 0.80 and 0.74 and 0.74, respectively). For predicting extracapsular extension, the 40% SUV model proved the best performing (0.77, respectively).

### 3.3. ^18^F-DCFPyl

Basso Dias et al. evaluated the value of PET and MRI radiomics for evaluation of ISUP GG1-2 vs. ISUP GG ≥ 3 [26]. The authors retrospectively studied 89 triple-positive (MRI-ADC- and T2w- and ^18^F-DCFPyl PET-positive) lesions in 86 treatment-naïve biopsy-proven prostate carcinoma patients (one patient had three lesions, two patients had two lesions, and the remaining patients had one lesion). PET tumor volumes were defined using a background threshold, a peak threshold, and a threshold at 40% and 70% of the SUVmax. On MRI, tumor contours were derived manually. Pet voxels size was 2.3 × 2.3 × 5 mm^3^. IBSI-compliant MRI- and PET-based radiomics features were extracted from the tumor volumes using the LIFEx software and subsequently used together with age, PSA levels, and lesions PROMISE (Prostate cancer Molecular Imaging Standardized Evaluation) classification for model-building following normalization. After building single models based on logistic regression, including features that maximized the Youden index, different combinations of models were generated, and ROC AUC values of these were models defined. The overall best model using two T2w-, two ADC-, and three PET-derived features had an AUC of 0.85, whereas the best-performing PET-only model including six features had an AUC of 0.79. Cysouw studied 76 patients with intermediate- to high-risk prostate carcinoma scheduled for robot-assisted radical prostatectomy with extended pelvic LN dissection that underwent pre-operative ^18^F-DCFPyl PET/CT [27]. The following pathology proven outcomes were dichotomized for machine learning-based classification, ISUP ≥ 4, the presence of ECE, pathology-proven LN involvement, and the presence of distant metastases. Tumor volumes were grown with thresholds varying from 50% to 70% on 2 × 2 × 2 mm^3^ voxel-size resampled images. Radiomic features (480 in total) were extracted from tumor volumes using the RaCaT software. Models were generated using an RF Classifier. Dimension reduction was performed using principal component analysis (retaining 95% of the variance), a recursive feature elimination approach, and an ANOVA-based univariate selection method. The radiomics-based machine learning models predicted LN involvement (AUC 0.86 ± 0.15), nodal or distant metastases (AUC 0.86 ± 0.14), ISUP ≥ 4 (0.81 ± 0.16), and ECE (0.76 ± 0.12). Dimension reduction had a limited effect on mean AUCs, and there was no apparent benefit of using one approach over the other. The most important textural features of ISUP prediction were zone size non-uniformity, zone distance non-uniformity, and gray-level variance, whereas intensity-based features were most important in the prediction of LNI. The use of a higher (70%) threshold and PVC proved beneficial for the prediction of various outcome variables.

## 4. Predicting Biochemical Recurrence

Following radical prostatectomy (RP) or prostate cancer radiation therapy, 27–53% of patients will develop a biochemical recurrence (BCR), defined as a PSA > 0.2 ng/mL, following RP and a PSA > post-RT nadir + 0.2 ng/mL [28]. Patients at high risk for BCR are ISUP groups 4 and 5 and pT3 patients with or without surgical margins who are currently offered adjuvant treatment [29]. Optimized models, including radiomics-derived information from PSMA-targeting PET examinations, could potentially provide a more accurate risk estimation. 

### ^68^Ga-PSMA-11

Papp et al. prospectively studied 52 patients that underwent baseline ^68^Ga-PSMA-11 PET/MRI and subsequently underwent radical prostatectomy. Tumor segmentation was performed using three-dimensional iso-count VOIs and corrected manually if required, resulting in 121 lesions in total [30]. Radiomic features with “very strong” or “strong” consensus values as of the IBSI guidelines, 442 in total, were extracted from the ^68^Ga-PSMA-11 (2 × 2 × 2 mm) PET, T2w, and ADC lesions by the MUW Radiomics engine. SUVmax, SUVmean, SUVpeak, and SUVtlg were merged with the 442 features, yielding a 446-long feature vector for each lesion. Feature reduction was subsequently performed by removing all features with correlation coefficients >0.75, leaving 80 features for further analysis. Models predictive for BCR (Mbcr) and overall patient risk (OPR, Mopr) were then built on random forest classifiers using training and validation lesion sets. During follow-up (mean 41 months), 36 patients presented with a BCR, and 50 had overall patient risk information available (high if BCR was positive or the node stage or metastases stage (clinical or pathological) were positive). The cross-validation performance revealed an average validation accuracy of 89% and 91% as well as an AUC of 0.90 and 0.94 for Mbcr and Mopr. Respective accuracies for the standard clinical model were 69% for BCR and 70% for OPR.

## 5. Machine Learning for Hotspot Classification 

Moazemi et al. studied 72 patients suffering from histologically proven prostate carcinoma that underwent ^68^Ga-PSMA PET/CT imaging, with Gleason scores ranging from 6 to 9 and serum PSA levels from 4 to 1840 ng/mL [31]. Sites of focal uptake beyond the local background were identified (2419 in total), delineated manually, and classified as malignant (1629) or physiologic/unspecific (790) by two experienced nuclear medicine physicians (gold standard). For each site, 40 PET-based and 44-CT based radiomic features were extracted using InterView Fusion software. Three feature groups were generated (PET only, CT only, and combined PET-CT), and five different ML algorithms, respectively, linear and radial basis function (RBF), polynomial kernel support vector machine, extra trees (ET), and random forest (RF), were applied to each subset. Overall, ET and RF yielded the highest accuracies for all three subsets (AUC ≥ 91%), with the combined use of PET and CT data yielding an accuracy of 98%. Using the same dataset as the validation group, the authors validated their results obtained using PET alone and an extended set of 77 extracted radiomic features in a validation cohort of 15 patients. In the latter group, 125 of the 128 lesions classified as pathologic were correctly identified (sensitivity of 97%), whereas specificity proved lower, 82%, respectively (overall accuracy of 0.98). The lower specificity was predominantly due to difficulties encountered by the AI algorithm in classifying sublingual and lacrimal glands (19/111 glands were identified as pathologic). Capobianco et al. developed and evaluated a multi-task convolutional neural network trained on ^68^Ga-PSMA-11 PET and CT information for the identification and anatomical classification of suspicious tracer uptake sites in the entire body [32]. The training set included image data of 123 consecutive patients suffering from prostate carcinoma referred for primary staging or for assessment of biochemical recurrence. The validation set consisted of a set of 50 patients referred for all kind of indications. Sites of increased uptake (5577 sites, of which 4520 were physiologic), were segmented using a 45% threshold of SUVmax. Inputs to the neural network were 13 parameters extracted after resampling to 3 mm isotropic voxels. The diagnostic accuracy of the neural network for separating malignant from benign sites of increased uptake was 80.4%.

## 6. Predicting Treatment Response and Overall Survival

^177^Lu-PSMA-617/I&T is being increasingly used for treating mCRPC patients; however, in 35–55% of patients, a biochemical response is not achieved. Biomarkers associated with response to ^177^Lu-PSMA therapy and outcome are thus of major clinical interest, given they could identify those patients that will respond favorably to this type of treatment upfront while avoiding unnecessary side effects to those who will not and reducing costs for society [33]. In this regard, Khurshid et al. assessed the predictive value of tumoral texture parameters in a series of 70 patients suffering from metastasized prostate carcinoma scheduled to undergo ^177^Lu-PSMA-therapy. Sixteen patients had previously also received ^223^Ra treatment [34]. For each patient, three bone metastases, three tracer avid lymph nodes, and liver, as well as other metastases when applicable, were manually delineated on ^68^Ga-PSMA-11 PET images. Subsequently, features were obtained from the histogram as well as from the normalized gray-level co-occurrence matrix (nGLCM). Based on ROC analysis and using changes in PSA induced by ^177^Lu-PSMA therapy (42 responders) as a gold standard, COV of the histogram, entropy, homogeneity, contrast, and size variation proved significantly correlated with changes in PSA, with entropy and homogeneity showing the lowest *p*-values, respectively, of 0.006 and 0.008. Moazemi et al. manually delineated 2070 pathological hotspots on ^68^Ga-PSMA-11 PET/CT images obtained in 83 prostate carcinoma patients, and for each hotspot 73 radiomic features (37 PET-based and 36 CT-based), were obtained [35]. Out of these features, the five radiomic features that correlated best with treatment-induced PSA changes were selected (respectively, PET SUVmin and PET-Correlation and CT-min, CT-coarseness and CT-busyness). When combined with a set of 14 clinical parameters and using an ML support vector machine classifier with radial base function, an AUC value of 0.80 was obtained for separating responders from non-responders. Using the same patient population and image analysis and the least absolute shrinkage and selection operator (LASSO) method, SUVmin and kurtosis were identified as the most relevant features related to overall survival. The latter two features were than used to form a radiomics signature (SUVmin × 0.984 + Kurtosis × −0.118), which, in the Kaplan–Meier analysis, proved significantly related to overall survival together with Hb, CRP, and ECOG status. Likewise, Roll et al. evaluated the predictive value of ^68^Ga-PSMA-11 PET/MRI for response to ^177^Lu-PSMA-therapy in a series of 21 mCRPC patients [36]. A threshold of 3.0 SUV was used to segment PSMA-positive lesions, out of which 162 first order logic features and 216 GLCM features were extracted in addition to standard SUV-metrics. Ten features, including three derived from PET, differentiated well between responders and non-responders. However, within the final model, features derived from MRI images proved more relevant than those derived from PET. Finally, Assadi et al. retrospectively studied 33 mCRPC patients that underwent ^177^Lu-PSMA treatment and in whom pretreatment ^68^Ga-PSMA-11 PET/CT scans were available [37]. All tracer-avid lesions, 2517 in total, identified on the ^68^Ga-PSMA-11 PET images were segmented semi-automatically using 3D Slicer software, and images were resampled to a voxel size of 2 × 2 × 2 mm^3^. Sixty-five features were extracted using the LIFEx software. Based on ICC analysis, all but GLZLM features proved acceptable. In addition to three clinical parameters, GLCM entropy (AUC 0.719) had an AUC value superior to 0.65 for predicting responders (≥50% decrease in PSA-value) from non-responders.

## 7. Discussion

Resolution recovery techniques have gained wide access in positron emission tomography imaging given they mitigate spatial resolution losses and related inaccuracies in quantification using a model of the system’s point spread function during reconstruction or post-processing. However, as shown by Fooladi et al. using ^68^Ga-PSMA-11 PET, their use significantly reduces the number of reproducible radiomic features as compared to standard reconstruction algorithms such as OSEM [12].

Interpolation allows for comparison of datasets spanning multiple centers with varying protocols using different voxel sizes, as well as the acquisition of isotropic rotation invariant voxels for three-dimensional feature extraction. In most of the studies reported, voxel size was reduced using interpolation, thus increasing the total number of voxels for radiomic feature extraction, which is of relevance given a minimum of 64 voxels is required for various texture features. The impact of interpolation on the robustness of radiomic features has been previously addressed by Whybra et al. for 18-fluoro-deoxyglucose (FDG) avid malignant lesions identified on ^18^F-FDG PET/CT [38]. Out of a series of 141 features, only 93 features proved robust to interpolation in their study. Data on the effect of interpolation on radiomic features extracted from PSMA-targeting-based PET-ligand avid prostate carcinoma are currently lacking, and its effect on predictive models is unknown and warrants further exploration.

As compared to FDG PET imaging, where region growing of 30–40% of the tumor’s SUV-max values has been shown previously to approximate the true tumor volume as assessed using histology, to the best of our knowledge, studies defining the threshold for accurate prostate tumor volume delineation have not been reported using PSMA-targeting ligands [39]. Thus, it is not clear whether radiomic features derived from prostate carcinoma lesions segmented using a semiquantitative technique, either the use of an SUV-cut-off value as applied in a limited number of studies or thresholding to 40% of the maximum SUV value as applied in the majority of studies reported, are representative for the total tumor volume. In fact, in a study by Zamboglou et al., primary prostate carcinoma volumes defined by region growing using a threshold of 40% proved significantly smaller than the tumor volume as defined using histology (median 7.3 mL versus 2.7 mL, *p* ≤ 0.05) [20]. This finding is surprising, given histology and especially tissue fixation and processing are known to shrink prostate carcinomata, requiring a compensation factor ranging from 1.14 to 1.55 depending on the histological technique used to obtain the true tumor volume [40,41]. Such a compensation factor was either not applied or reported in the study by Zamboglou et al. [20]. Based on the findings by Cysouw et al., the use of a higher (70%) threshold is likely to be more accurate given, in their study, this threshold proved more beneficial for prediction of various histological outcome variables in prostate carcinoma when compared to a 40% or 50% threshold [27]. Of interest, a number of authors have also extracted radiomic features from a half-glandular or a full-glandular segmented prostate. As opposed to segmenting the primary tumor only, this approach offers a number of advantages. First, automatic and semi-automatic methods for segmentation of the full prostate are already available and commonly used, e.g., in a radiotherapeutic setting. Second, when the standard PET/CT camera settings include the use of isotropic voxels, interpolation, which reduces feature reproducibility, may be avoided given the use of the entire prostate for radiomic features extraction averts the risk of insufficient voxel-numbers. Furthermore, as shown by Zambouglo et al. features extracted from the entire prostate subtracted by the tumor volume may allow for the identification of missed small but clinically significant prostate carcinoma in a relevant number of patients [21].

Importantly, as shown by Kendrick et al., radiomic features derived from the same malignancy using different PSMA-targeting ligands are not interchangeable [15]. In their study, the robustness of ^18^F-PSMA-1007 PET-derived features proved overall lower than that of ^68^Ga-PSMA-11 PET-derived features, with inter-tracer robustness being the worst of all. While both ligands have been reported to have a comparable affinity, the blood clearance rate differs significantly, with 18F-PSMA-1007 being more slowly cleared, likely resulting in a higher blood pool contribution to the radiomics features extracted. Furthermore, as suggested by other studies reporting on a higher number of false-positive bone findings using ^18^F-labelled PSMA-targeting agents when compared to ^68^Ga-PSMA-11, dehalogenation, the degree of which is patient and time variable, is also likely to contribute to the higher variability reported. A poor robustness was also reported for radiomic features extracted in a test-retest setting from ^18^F-DCFPyL [16].

As addressed previously, Gleason scores derived from biopsy and after resection are discordant in a large number of prostate carcinoma patients. Radiomics analysis and machine learning applied to PSMA-radioligand avid primary lesions may prove of clinical utility in this regard given the available studies have suggested that it might be better performing than biopsy-based GS scoring and thus could serve as an alternative for non-invasive GS characterization in the future [19,27]. Furthermore, one study suggested that radiomic signatures may allow for the prediction of biochemical recurrence with a net benefit for clinical utilization [30]. How this finding can be incorporated into more personalized therapy schemes, e.g., the administration of adjuvant therapy, will need to be evaluated in prospective trials. 

A limited number of studies further suggest that machine learning based on PET/CT radiomics features can differentiate increased tracer uptake on PSMA-targeting radioligand PET/CT examinations in malignant versus physiological or unspecific uptake, paving the way for a fully automated image reading in nuclear medicine [31,32].

As for prediction to treatment outcome following ^177^Lu-PSMA therapy and overall survival, the results of a limited number of studies, including a small number of patients, have reported promising results on radiomics and machine learning applied to PSMA-targeting radioligand PET/CT images for this purpose. Its added value as opposed to the clinical parameter, however, warrants further exploration in larger datasets of patients, including multivariate analysis.

While promising, the currently published studies in the field of radiomics and PSMA-targeted PET suffer from various shortcomings. First, the quality of their reporting may be significantly improved as suggested by the recent Checklist for Evaluation of Radiomics Research (CLEAR) guidelines endorsed by the European Society of Medical Imaging Informatics (EuSoMII) [10,11]. EuSoMII more recently also endorsed the utilization of a methodological Radiomics Score (METRICS), a quality scoring tool for radiomics research that takes into consideration the study design, imaging data, imaging processing and feature extraction, and metrics, as well as comparison and testing [11]. More specifically, for references [20,21,22,23,24,25,26,27] (see also Table 2) and [30,31,32,33,34,35,36,37], including a histological or a well-validated clinical gold standard, METRICS scores obtained were either moderate or good, respectively, in 12 studies and 4 studies, leaving room for improvement. In several studies, radiomic features known to bear a poor reproducibility were included, whilst such features are not likely to be informative. Also, related to the software used, the range of number of features extracted varies widely form one study to another, as well as the number of features used for modeling. The higher the number of features included, the higher the risk of model over-fitting is. The same problem exists when including low numbers of patients, as was the case in some studies. Finally, in most of these studies, internal validation was performed using cross-validation and not by splitting up the study sample at the beginning of the study, likely related to the small number of patients included. Furthermore, external validation was only performed in one study, and most studies did not systematically make available their data via open sourcing. 

## 8. Conclusions

This review determined that while promising, the limited available data on the use of radiomics and PSMA-targeted PET in prostate carcinoma do not currently allow for their implementation in routine clinical practice. Studies performed according to recently published guidelines and checklists, offering full transparency, that are clinically relevant, including large enough datasets as well as external validation, are mandatory.

## Figures and Tables

**Table 1:** Methodological studies.

Authors	Tracer	Nb of Lesions/Pts	Methodology	Results
Fooladi et al. [12]	^68^Ga-PSMA-11	30/8	Q.Clear vs. OSEM/41%threshold/LIFEx (129 features)	OSEM yields more reproducible results than Q.Clear
Pasini et al. [13]	^18^F-PSMA-1007	78/78 (primary)	Manual vs. region growing vs. thresholding, pyradiomics (1781 features)	GLCM features most reproducible, shape features least reproducible
Dutta et al. [14]	^68^Ga-PSMA-11	142/142 (primary)	Automated versus manually delineated, pyradiomics (1037 features)	19% of features proved reproducible
Kendrick et al. [15]	^68^Ga-PSMA-11 and ^18^F-PSMA-1007	18/75	Intra- ^68^Ga-PSMA-11 (5 pts), intra-^18^F-PSMA-1007 (5 pts) and inter-tracer (8 pts), threshold-based, Pyradiomics (107 features)	Most reproducible features on intra-^68^Ga-PSMA-11—inter-tracer poorly reproducible
Werner et al. [16]	^18^F-DCFPyl	21/230	Manual delineation, Interview software (29 features)	Only entropy and homogeneity proved reproducible

OSEM = ordered subset expectation maximization, GLCM = gray-level co-occurrence matrix.

**Table 2:** Characterization and discrimination.

Authors	Tracer/M-Score	Nb of ppc Studied	Methodology and Software Used	Results
Zamboglou et al. [20]	^68^Ga-PSMA-11 /moderate	20/40 pts	2 × 2 × 2 mm^3^ voxels, wavelet filtered (ppc (40%), entire prostate (CT), delta both volumes//versus histology (volumetry)/in-house MATLAB software	ppc volume (40%) significantly smaller than histologic volumes/QSZHGE best performing feature
Zamboglou et al. [21]	^68^Ga-PSMA-11 /Good	20/52 pts	2 × 2 × 2 mm^3^ voxels, locally binary filtering (LBP), pyradiomics	Best-performing features were LBP-SZNUN and LBP-SAE
Yi et al. [22]	^68^Ga-PSMA-11 /Good	64/36	Prostate manually segmented, 3 × 3 × 3 mm^3^, pyradiomics 3.1.0	RF model (10 most perfoming features), AUC = 0.903
Ghezzo et al. [23]	^68^Ga-PSMA-11 /Moderate	43	Prostate manually segmented, 2 × 2 × 2 mm^3^ voxels, pyradiomics/ISUP prediction	LR, SVM, K-nearest neighbor, best-performing model 87.6% acuracy versus 85.9% for biopsy
Solari et al. [24]	^68^Ga-PSMA-11 /Moderate	101	Prostate manually segmented/fuzzy locally adaptive Bayesian based segmentation, voxel-size not mentionned, pyradiomics	SVM, ACC 87%
Yao et al. [25]	^18^F-PSMA-1007 /Moderate	173	Region growing 30%/40%/50%/60%, 2 × 2 × 2 cm^3^, LIFEx software	LR-based models (50%, AUC 0.82)
Basso Dias et al. [26]	^18^F-DCFPyl/Moderate	89	2.3 × 2.3 × 5 mm^3^ voxels, 40% and 70%, LIFEx software, PET and MRI	LR-based models (AUC 0.85)
Cysouw et al. [27]	^18^F-DCFPyl/Good	76	2 × 2 × 2 mm^3^ voxels, 50% and 70%, RaCaT software	RF-models, AUC (0.86 (LN involvemnet, 0.86 distant metastases, ECE (0.76)

ppc = primary prostate carcinoma, ISUP = international society of urological pathology, LR = logistic regression, SVM = support vector machine, ACC = accuracy, AUC = area under the curve, ECE = extra capsular extension, LN = lymph node. In the first three studies, two patient cohorts were included. M-score = METRICS score.
